# Development of Bigels Based on Date Palm-Derived Cellulose Nanocrystal-Reinforced Guar Gum Hydrogel and Sesame Oil/Candelilla Wax Oleogel as Delivery Vehicles for Moxifloxacin

**DOI:** 10.3390/gels8060330

**Published:** 2022-05-24

**Authors:** Hamid M. Shaikh, Arfat Anis, Anesh Manjaly Poulose, Niyaz Ahamad Madhar, Saeed M. Al-Zahrani

**Affiliations:** 1 SABIC Polymer Research Centre, Department of Chemical Engineering, King Saud University, P.O. Box 800, Riyadh 11421, Saudi Arabia; aarfat@ksu.edu.sa (A.A.); apoulose@ksu.edu.sa (A.M.P.); szahrani@ksu.edu.sa (S.M.A.-Z.); 2 Department of Physics and Astronomy, College of Sciences, King Saud University, P.O. Box 2455, Riyadh 11451, Saudi Arabia; nmadhar@ksu.edu.sa

**Keywords:** date palm-derived cellulose nanocrystal, guar gum hydrogel, sesame oil, candelilla wax, oleogel, drug delivery

## Abstract

Bigels are biphasic semisolid systems that have been explored as delivery vehicles in the food and pharmaceutical industries. These formulations are highly stable and have a longer shelf-life than emulsions. Similarly, cellulose-based hydrogels are considered to be ideal for these formulations due to their biocompatibility and flexibility to mold into various shapes. Accordingly, in the present study, the properties of an optimized guar gum hydrogel and sesame oil/candelilla wax oleogel-based bigel were tailored using date palm-derived cellulose nanocrystals (dp-CNC). These bigels were then explored as carriers for the bioactive molecule moxifloxacin hydrochloride (MH). The preparation of the bigels was achieved by mixing guar gum hydrogel and sesame oil/candelilla wax oleogel. Polarizing microscopy suggested the formation of the hydrogel-in-oleogel type of bigels. An alteration in the dp-CNC content affected the size distribution of the hydrogel phase within the oleogel phase. The colorimetry studies revealed the yellowish-white color of the samples. There were no significant changes in the FTIR functional group positions even after the addition of dp-CNC. In general, the incorporation of dp-CNC resulted in a decrease in the impedance values, except BG3 that had 15 mg dp-CNC in 20 g bigel. The BG3 formulation showed the highest firmness and fluidity. The release of MH from the bigels was quasi-Fickian diffusion mediated. BG3 showed the highest release of the drug. In summary, dp-CNC can be used as a novel reinforcing agent for bigels.

## 1. Introduction

In recent years, bigel-based delivery systems have been proposed as novel biphasic systems [1]. Such systems are developed by mixing two types of gelled systems of different polarities, namely hydrogels and oleogels, under a controlled temperature [2]. Hydrogels are gelled systems that are hydrophilic systems of aqueous solvent [3], while oleogels are hydrophobic gelled systems of edible oils [4]. The mixing of the aforesaid gelled systems leads to the formation of biphasic systems, which are structurally similar to emulsions [5]. However, unlike emulsions, bigels are semisolid in nature [6]. Depending on the distribution of the gelled systems, bigels are categorized either as oleogel-in-hydrogel or hydrogel-in-oleogel [2]. Moreover, some authors have reported the formation of bi-continuous bigel [2,6]. This type of bigel does not have either a clearly dispersed or continuum phase. The main advantage of bigels is their improved stability and shelf-life [7]. The composition of the constituting oleogels and hydrogels alters the properties of the bigels. Also, the proportion of the oleogel and the hydrogel can govern their properties [8,9]. Since the bigels consist of both hydrophobic (oleogels) and hydrophilic (hydrogels) components, they are a good candidate for delivering both hydrophobic and hydrophilic bioactive agents, either individually or simultaneously [10,11,12]. Due to the versatile properties of bigels, they have been used for pharmaceutical, food, and cosmetic applications.

Oleogels are gel-based systems of lipids and oils [13,14]. These gels are hydrophobic, unlike hydrophilic hydrogels. In recent times, the use of oleogels in food and pharmaceutical applications has received much attention as a solid fat replacer/alternative [15]. The use of oleogels helps reduce the saturated fatty acid content and consequently increases the unsaturated fatty acid content in food products [16,17,18]. The oleogels entrap oils within a network structure of the gelators, which are hydrophobic. One of the common types of gelators are vegetable waxes (e.g., mango butter, cocoa butter, sunflower wax, and candelilla wax) [18,19,20]. Vegetable wax-based oleogels are prepared by the direct dispersion method, which is one of the most common and easiest methods of oleogel preparation [21]. Since vegetable waxes are rich in fat molecules, fat crystals are formed within the gelator network of oleogels during the synthesis process [19,20].

In the present study, candelilla wax (CW) and sesame oil (SO)-based oleogel will be used as the model oleogel. CW is extracted from the leaves of candelilla shrubs (*Euphorbia cerifera* and *Euphorbia antisyphilitica*; family: Euphorbiaceae) [22]. The shrub is mainly found in the region of northern Mexico and the southwestern United States [23,24]. CW is yellowish-brown in color and has a melting point of 62–70 °C [25]. Due to its vegan origin, it has been proposed for food applications. Oleogels of CW have been explored in recent times as saturated fat replacers [26]. SO is extracted from sesame seeds (*Sesamum indicum*; family: Pedaliaceae) [27]. The oil has a high linoleic acid and oleic acid content, which combined constitute nearly 80% of the total fatty acid content [28,29]. It is one of the most widely used cooking oils across the globe and has also been used to develop various food products.

Hydrogels are polymeric networks that entrap water molecules. In recent years, polysaccharides have been explored for developing hydrogels. Polysaccharides are naturally-occurring polymers and are inherently biocompatible. Among various polysaccharides (e.g., guar gum, alginic acid, chitosan, gum tragacanth), guar gum (GG) is one of the most widely used polysaccharides. GG is obtained from the seed of the guar plant (*Cyamopsis tetragonolobus*; family: Leguminosae) [30,31]. The polysaccharide is composed of D-mannopyranose (M) monomer units [32,33]. This natural polymer has been used in the food, pharmaceutical, and cosmetics industries for a long time. Researchers have proposed altering the properties of GG-based polymeric architectures with cellulose nanocrystals (CNCs) [34]. The inclusion of CNC into GG-based formulations help to tailor the properties of the formulations.

Similarly, nanocellulose has a remarkable skeletal structure, due to its numerous hydrophilic functional groups and nano size effect, which allows it to maintain the hydrogel’s three dimensional structure to a large extent while maintaining the moisture content [35]. Also, a high degree of polymerization and a large surface area to volume ratio result in increased drug loading and binding capacity for drug release. However, a variety of factors, such as cellulose source, isolation strategy, size, and shape determine the optimum performance of nanocellulose [36]. Cellulose nanocrystals can be obtained from agricultural waste and used in a range of applications. In earlier work, we isolated cellulose nanocrystals from date palm (dp-CNC) tree residues. Furthermore, the reinforcing influence of the dp-CNCs on the bigels has not been studied yet. Therefore, it is reasonable to investigate the potential of this nanocellulose to tailor the properties of bigels.

Maharana, V. et al. [37] have proposed altering the properties of the filled hydrogels by reinforcing the dispersed phase. In the study, the authors developed gelatin-tamarind gum-filled hydrogels (also known as bigels), wherein the tamarind gum was reinforced with carbon nanotubes. The authors demonstrated that the reinforcement of the dispersed phase significantly affected the properties of the filled hydrogels. These carbon nanotubes were found to improve associative interaction among hydrogel components and to maintain the architecture of the hydrogels. Accordingly, in this study, we developed a GG hydrogel-in-CW/SO oleogel bigel. The GG hydrogel was reinforced with varying amounts of date palm-derived CNC (dp-CNC). The synthesis of the bigels was performed by the facile mixing approach. The properties of the bigels were then analyzed by microscopic, FTIR spectroscopic, XRD, impedance spectroscopic, and texture analysis methods. The developed bigels were also explored as carriers for Moxifloxacin (MH).

Moxifloxacin (MH) is categorized under the quinolone group of antibiotics. The drug is a broad-spectrum antibiotic and is used to treat a wide variety of bacterial infections. Accordingly, it has been used to treat several bacteria-induced diseases. Some of the diseases where MH is used as a drug of choice include respiratory tract infections, conjunctivitis, tuberculosis, endocarditis, and pneumonia. Though the drug’s elimination half-life is ~12 h, most of the drug is excreted through feces or urine. Accordingly, there is a need to design controlled drug delivery systems that can prolong the release of the drug and hence improve bioavailability. In this regard, bigels have been proposed as a novel drug delivery system by many researchers. The knowledge gathered through this study could allow the scientific community to understand how the reinforcement of the internal phase of bigels with CNC affects the properties of the bigels.

## 2. Results and Discussion

### 2.1. Microscopic Evaluation

**Figure 1:**
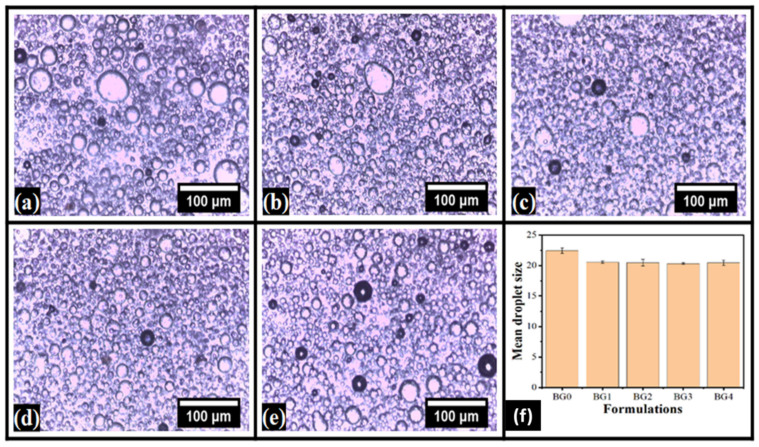
Bright-field micrographs of the bigels. (**a**) BG0, (**b**) BG1, (**c**) BG2, (**d**) BG3, and (**e**) BG4. (**f**) Variation in the mean droplet size.

The analysis of the optical micrographs of bigels can provide information about the dispersion of the dispersed phase (Figure 1). Observation of the micrographs suggest the presence of globular phases within a continuum phase. As the proportion of the hydrogel phase was only 25%, it can be expected that the dispersed phase would be the hydrogel [38]. Consequently, the continuum phase would be the oleogel phase. It can be seen that BG0 (Figure 1a) showed a wide distribution in the size of the globular phases. A large number of bigger globular structures could be observed in BG0 (Figure 1a). As dp-CNC was incorporated in BG1 (Figure 1b), there was a drastic reduction in the size of the globular structures. However, some bigger size droplets could be seen. A corresponding increase in the dp-CNC content in BG2 and BG3 (Figure 1c,d), resulted in smaller globular structures, respectively. Apart from the formation of smaller globular structures, there was increased homogeneity. It can be observed that the globular size distribution was highly homogenous in BG3 (Figure 1c). A further increase in the dp-CNC content in BG4 (Figure 1e) caused a slight increase in the size of the globular structures and increased size distribution. Additionally, it can also be observed that some of the globular structures in BG0, BG1, and BG4 (Figure 1a,b,e) were apparently deformed. Nevertheless, the globular structures in BG2 and BG3 (Figure 1c,d) were relatively non-deformed. The variation in the mean droplet size (Figure 1f) suggested that the addition of dp-CNC reduced the droplet size of the internal phase. The difference in the droplet size of the internal phase among the dp-CNC-containing formulations was not significant. The droplet size was calculated manually using ImageJ software.

**Figure 2:**
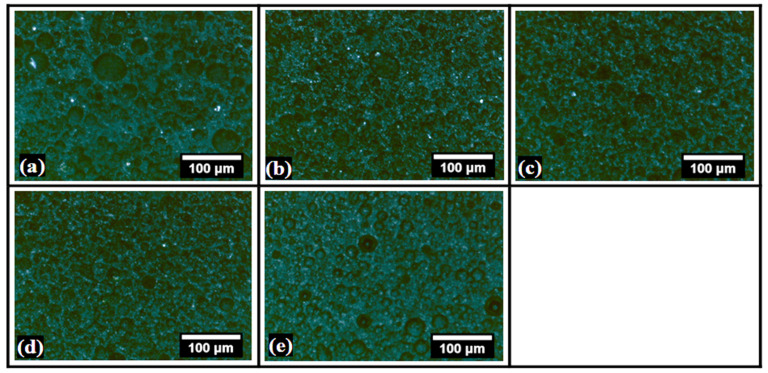
Polarized light micrographs of the bigels. (**a**) BG0, (**b**) BG1, (**c**) BG2, (**d**) BG3, and (**e**) BG4.

The polarized light micrographs of the bigels are presented in Figure 2. It can be observed from the micrographs that the dispersed phase was darker than the continuum phase, samples BG0-BG4 (Figure 2a–e). It is well-established that fat crystals appear brighter under polarizing conditions [39]. This has been attributed to the ability of fat crystals to diffract light. The diffracted light can be captured by polarizing microscopy. On the other hand, hydrogels are amorphous structures and appear as dark objects under polarizing microscopy, i.e., samples BG4 (Figure 2e). Analyzing the microarchitecture confirms that the dispersed phase was the hydrogel phase, while the continuum phase is composed of the oleogels. Further, the polarizing micrographs also corroborated the observations from the optical micrographs. Therefore, it can be concluded that the addition of dp-CNC within the inner phase of the hydrogel-in-oleogel bigels can help to tailor the size and distribution of the dispersed phase. It could be further seen that dp-CNC helped form an un-deformed dispersed phase of the hydrogels in bigels. The alteration in the properties of the dispersed phase occurs in a concentration-dependent manner.

### 2.2. Colorimetry

**Figure 3:**
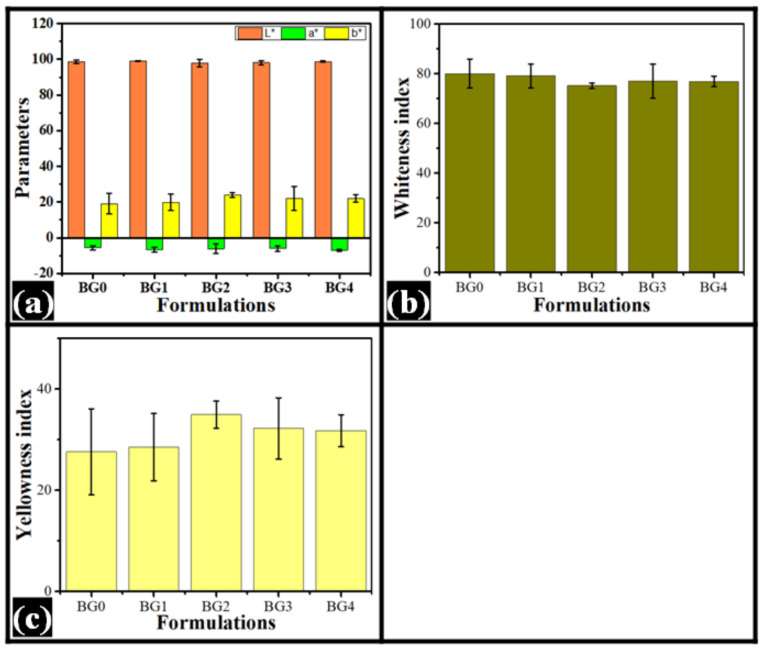
Colorimeter profile of bigels. (**a**) CIELab color parameters, (**b**) Whiteness index, (**c**) Yellowness index.

The results of the colorimetric analysis have been compiled in Figure 3 and Table 1. The color analysis was carried out in the CIELab color plane, wherein L *, a *, and b * values are obtained [40]. The main advantage of this color model is that it encompasses the entire range of human visual color perception [41]. The “L *” parameter is independent of chromaticity information. It basically represents the perpetual lightness of the samples [42]. From the results, it can be observed that the average “L *” value was in the range of 97.76 and 99.07. However, the “L *” values were not statistically significant from each other. On the other hand, “a *” and “b *” values are chromic parameters, wherein “a *” and “b *” parameters represent green-red and blue-yellow opponent colors, respectively [43,44]. The negative values of “a *” and “b *” represent green and blue colors. The positive values of “a *” and “b *” represent red and yellow colors. Analysis of the “a *” and “b *” values suggest the presence of green and yellow hues within the system. The variation in the values of the parameters was not statistically significant. Apart from the aforesaid primary color parameters, derived parameters, namely whiteness index (WI) and yellowness index (YI), were also obtained from the instrument [45]. The WI was in the range of 75% and 80%, while the YI was in the range of 27% and 35%. There was no significant difference in the WI and YI values of the formulations. In summary, the formulations were highly reflective in nature with predominantly green and yellow hues. The addition of dp-CNC did not alter the color of the formulations.

### 2.3. FTIR Spectroscopy

**Figure 4:**
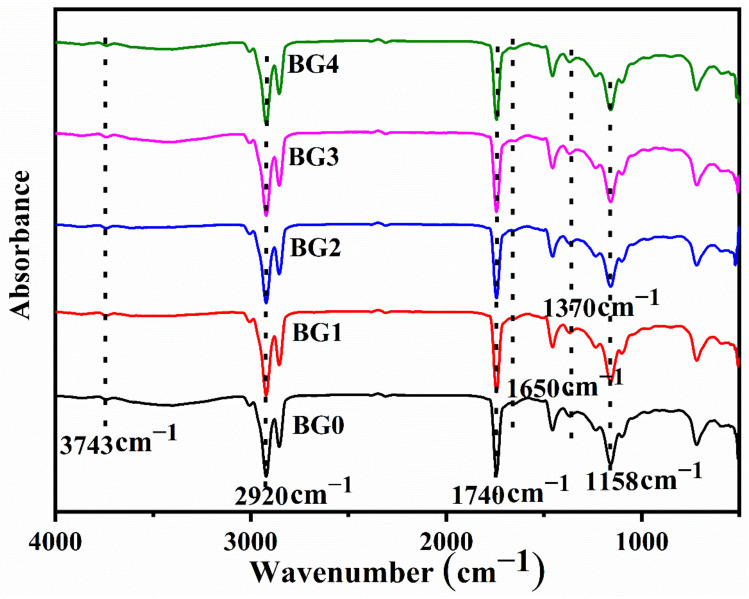
FTIR spectra of the bigels.

The FTIR spectrum provides useful information about the presence of potential functional groups and interactions within them in bigel formulations. The FTIR spectra of the bigels have been provided in Figure 4. The analysis of the functional groups is mainly done in the functional group region. The FTIR spectra of the bigels showed that there was a sharp peak at 1740 cm^−1^. This peak is associated with the -C=O stretching vibrations of the esters that are present in lipids and fatty acids [46].

The presence of dual peaks at 2854 cm^−1^ and 2920 cm^−1^ is due to the sp3 hybridized carbon atom C-H stretching, which was in abundance in SO, CW, GG, and dp-CNC [47]. The broad peak at 3425 cm^−1^ was due to the O-H stretching vibrations due to hydrogen-bonded hydroxyl groups [48]. Further, another peak was observed at 3743 cm^−1^ that can be explained by the O-H stretching of the free hydroxyl groups [49]. The hydroxyl functional groups were in abundance in SO, CW, GG, and dp-CNC. Apart from these major peaks in the functional group region, a couple of minor peaks were also observed. The minor peak at 1650 cm^−1^ was due to the N-H bending of primary amines that are present in GG. Apart from the peaks mentioned in the functional group region, some additional peaks at 1370.7 cm^−1^, 1234.6 cm^−1^, 1158.4 cm^−1^, and 1102.7 cm^−1^ were observed. These peaks are considered to be in the fingerprint region. These peaks can be associated with different vibration modes of methyl (-CH_3_) and methylene (-CH_2_) groups in SO, CW, GG, and dp-CNC [50]. In summary, all the formulations (control and dp-CNC-containing bigels) showed peaks precisely at the exact location. This indicates the presence of similar types of interactions among all the formulations, including the control sample. However, there were some variations in the intensity levels.

### 2.4. Analysis of Impedance

**Figure 5:**
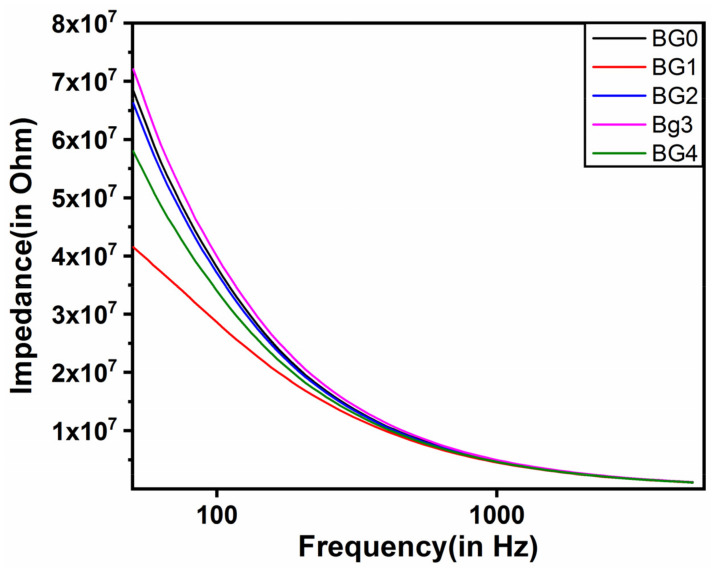
Impedance profiles of the bigels in 50 and 5000 Hz frequency range.

The impedance profiles of the bigels are provided in Figure 5. The impedance profiles showed typical capacitive behavior; wherein there is a higher impedance in the low-frequency range that reduces to a basal level in the high-frequency range [51]. The analysis of the impedance profiles suggested that the control bigel (BG0) was relatively higher than the dp-CNC-containing bigels, except BG3. This indicated that the addition of dp-CNC in the bigels reduced the impedance of the bigels in general. Among the dp-CNC-containing bigels, BG1 had the lowest impedance. In other words, the electrical conductivity of BG1 was the highest. The impedance value was correspondingly increased in BG2 and BG3, respectively, with increase in dp-CNC content. In fact, the impedance of BG3 was the highest. Thereafter, a corresponding fall in the impedance value was observed in BG4, which contained the highest amount of dp-CNC.

The observed impedance can be related to the microstructure of the bigels. From the micrographs, it was observed that BG3 showed smaller and homogenous droplets. As the amount of the hydrogel was the same in all the bigels, the smaller droplets resulted in the presence of more droplets. These droplets acted as numerous capacitive elements, thereby resulting in the highest impedance of BG3. The impedance of the other bigel formulations can also be related to droplet size and homogeneity.

### 2.5. Stress Relaxation Studies

**Figure 6:**
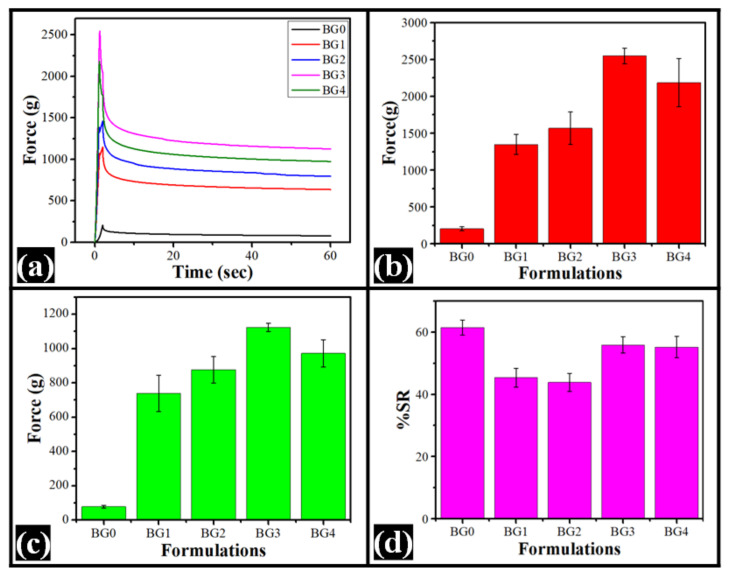
Stress relaxation study of the bigels. (**a**) SR profile, (**b**) F_0_ profile (average ± standard deviation), (**c**) F_60_ profile (average ± standard deviation), and (**d**) % SR profiles (average ± standard deviation).

The stress relaxation (SR) profiles and their parameter values are presented in Figure 6 and Table 2. The F_0_ value, maximum force attained in the SR profile, of BG0 was significantly lower than the dp-CNC containing formulations (*p* < 0.05). This could be associated with the reinforcing properties of CNC. Among the dp-CNC containing formulations, there was an increase in the F_0_ values from BG1 to BG3. Subsequently, an increase in the dp-CNC content decreased the F_0_ value in BG4. However, the differences in the F_0_ values among BG1 and BG2, BG2 and BG4, and BG3 and BG4 were statistically insignificant (*p* > 0.05). The F_60_ values, the minimum force value at the end of the relaxation process, followed a trend similar to F_0_ values.

The %SR value provides information about the fluidity (or elasticity) of formulations [52,53]. A higher %SR value indicated higher fluidic nature of the formulations. The analysis of the %SR values suggested that BG0 was highly fluidic in nature and had the highest %SR value. However, the %SR value of BG0 was similarly valued with that of BG3 and BG4 (*p* > 0.05). The %SR value of BG1 and BG2 was the lowest (*p* > 0.05). This suggested that the elastic component of BG1 and BG2 was higher than all the other formulations (*p* < 0.05). Interestingly, an increase in the dp-CNC content in BG3 and BG4 significantly increased the fluidic component within the bigels that were similarly to each other to control (BG0) (*p* > 0.05). In brief, BG3 had the highest firmness and, at the same time, improved fluidity.

### 2.6. Drug Release Study

The Moxifloxacin HCI is a new fluoroquinolone antibacterial agent that works against both gram-positive and gram-negative bacteria. It has better activity against anaerobes and gram-positive bacteria (such as streptococci, staphylococci, and enterococci) than ciprofloxacin [54]. Moxifloxacin HCI is used to treat bacterial infections of the respiratory tract, including community-acquired pneumonia, sinusitis, and acute exacerbations of chronic bronchitis [55]. The FDA authorized moxifloxacin HCI ophthalmic solution in April 2003 for the treatment of bacterial conjunctivitis caused by susceptible species [56]. An improved antibacterial activity was reported from chitosan/β-glycerophosphate in situ-forming thermo-sensitive hydrogel loaded with moxifloxacin HCI (0.25% *w*/*v*) compared to moxifloxacin HCI solution (0.5% *w*/*v*) [57]. Accordingly, formulations of 0.25% of moxifloxacin HCI were developed in this study.

**Figure 7:**
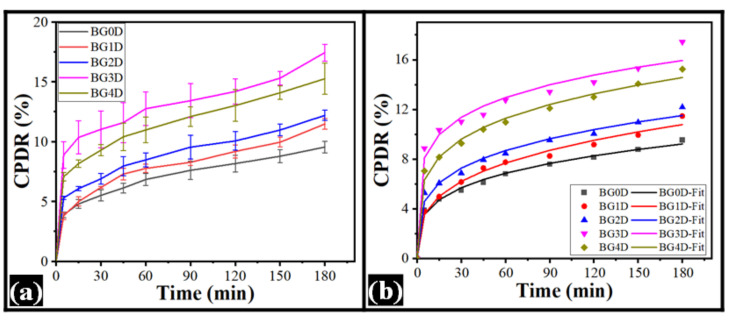
Drug release profile of bigel formulations. (**a**) CPDR profile and (**b**) KP model of CPDR.

The release profiles of the drug moxifloxacin HCl from the formulations are provided in Figure 7. It can be seen from the drug release profiles that an increase in the dp-CNC content correspondingly increased the CPDR (cumulative percent drug release) till BG3D. However, there was a subsequent decrease in the CPDR values from BG4D. Among the dp-CNC-containing formulations, the CPDR values were in the same order as that of the impedance values in the low-frequency region. Statistically, the CPDR values of the dp-CNC containing formulations were significantly higher than the control (*p* < 0.05). This is suggestive of the fact that the addition of dp-CNC promoted the release of the drug. Further, the differences in the CPDR values at the end of the experiment of BG1D and BG2D, BG2D and BG4D, and BG3D and BG4D were statistically similar to each other (*p* > 0.05). Overall, the enhanced drug release, due to the addition of dp-CNC, could be explained by faster diffusion of the drug molecules within the hydrogel matrices, which improved water absorption capacity, due to dp-CNC, may have promoted.

The CPDR profiles were then fitted to the Korsmeyer-Peppas (KP) model (Equation (1)) [58]. The parameters of the KP model are formulated in Table 3. It can be observed that the diffusion factor (K) of BG0D, with respect to BG1D and BG3D, was statistically insignificant (*p* > 0.05). The incorporation of dp-CNC resulted in increase in the ‘K’ value in a concentration-dependent manner. However, the ‘K’ values of BG1D and BG3D, BG2D and BG3D, and BG3D-BG4D were statistically insignificant (*p* > 0.05). The diffusion exponent (n) values were less than 0.45, suggesting quasi-Fickian diffusion of the drug molecules during the drug release process:

(1) $$\mathrm{CPDR}=K\times t^{n}$$

where CPDR is cumulative percent drug release, K is the diffusion factor, t is the time of sampling, and n is the diffusion exponent.

## 3. Conclusions

GG hydrogel and SO/CW oleogel-containing bigels were developed using an easy and facile method in the present study. Dp-CNC was then incorporated within the bigel in varied amounts to alter the physical and biochemical properties of the bigel. It was found that the developed bigels were smooth and stable. The bigels were yellowish-white in color. The formation of biphasic formulations was confirmed by bright field and polarizing microscopy. The IR spectra suggested that there was no change in the interactions of the bigel components after the addition of dp-CNC. The incorporation of dp-CNC improved the firmness of the formulations in a composition-dependent manner. This was associated with the reinforcing property exerted by CNCs. At lower dp-CNC content, the bigels contained more elastic components, while the fluidic component was more predominant when the dp-CNC content was on the higher side. The release of the drug MH was diffusion-mediated and followed quasi-Fickian release kinetics. In closing, it was observed that dp-CNC could be explored as a novel reinforcing and drug release agent, which could be used to develop delivery systems for antimicrobial agents. Finally, the summary of the key parameters is tabulated in Table 4.

## 4. Materials and Methods

### 4.1. Materials

Candelilla wax (CW, BiOrigins, Hampshire, UK), and Sesame oil (SO, Massy Cedex, France) were procured from the local hypermarket. Guar gum was used from Scharlab, Barcelona, Spain. Disodium hydrogen phosphate, and potassium dihydrogen phosphate were procured from Merck, Darmstadt, Germany. Date palm-derived CNCs were synthesized in our laboratory as per the method described earlier [36]. This has nanoparticles with sizes ranging from 26 nm to 61 nm, a negative zeta potential of −35 mV, and 89% crystallinity. Also, double distilled water was used throughout the study.

### 4.2. Preparation of the Formulations

#### 4.2.1. Preparation of the Oleogel

The critical gelation concentration of CW for inducing gelation of SO was initially determined. For this purpose, a specified amount of CW was added to SO. The mixture was then heated at 65 °C for 30 min to induce the dissolution of CW in SO. Then, the hot mixture was kept at 25 °C for 120 min to allow the gelation process to complete. The amount of CW was varied to determine the CGC. The CGC of CW for SO was found to be 7%. The oleogel prepared at CGC was used for further studies.

#### 4.2.2. Preparation of the GG Hydrogels

GG hydrogel was prepared by dispersing 1 g of GG in 99 g of water that was kept stirring at 800 RPM. The dp-CNC loaded GG hydrogels were prepared by dispersing a sufficient amount of dp-CNC in water, followed by the addition of GG in water. The amount of dp-CNC was added so as to maintain the dp-CNC content in the formulations, as mentioned in Table 5.

#### 4.2.3. Preparation of the Bigels

Specified amounts of GG hydrogels (60 °C) were slowly added to the oleogel (60 °C), which was kept on homogenization at 800 RPM. The homogenization was further continued for 20 min. Then, the mixture was kept at room temperature (25 °C) for 1 h. The reduction of the temperature of the mixture induced gelation to form bigels. The drug-containing bigels were prepared by incorporating the drug (moxifloxacin hydrochloride; moxifloxacin HCl) within the GG hydrogel phase. The composition of the bigels is presented in Table 5.

### 4.3. Characterization

The microstructures of the bigels were initially visualized using an optical bright field microscope. Subsequently, polarized microscopy was performed using the same microscope, which was attached with an in-house built polarizer and an analyzer. The microscope was fitted with an ICC50-HD camera for imaging. The prepared bigels were then subjected to colorimetric analysis using a reflective colorimeter (X-Rite Ci7600 spectrophotometer, Grand Rapids, MI, USA).

The functional group identification and their interactions were studied using an FTIR (Fourier Transform infra-red spectroscope Nicolet iN10, Thermo Scientific, Winsford, UK) working in the ATR (Attenuated total reflectance) mode. The analysis was performed in the wavenumber range of 3500 cm^−1^ to 500 cm^−1^. An average of 32 scans was used for the final spectra. The resolution of the instrument was 4 cm^−1^. The impedance analyses of the bigels were carried out using an impedance analyzer (Digilant, Pullman, WA, USA). The analysis was carried out through the parallel plate capacitive method. Briefly, the stainless electrode system, consisting of two circular parallel plates (diameter: 1 cm, distance between plates: 1 cm), was inserted into the sample. Then, the impedance was measured in the frequency range of 50 and 5000 Hz.

The viscoelastic properties of the bigels were analyzed by the stress relaxation (SR) study. The SR study was carried out using a mechanical tester (TA HD-plus, Stable Micro Systems, Haskmere, UK). The study was conducted using a 30 mm flat probe. The probe was allowed to penetrate the sample by 1 mm after sensing a force of 5 g. The initial force value at this position was regarded as F_0_. Thereafter, the probe remained at the same location for 1 min. The force at the end of this time was regarded as F_60_. During this time, the variation in the force profiles was recorded and consequently analyzed. The percentage SR (%SR) was calculated using Equation (1):

(2) $$\%\mathrm{SR}=\frac{F_{0}-F_{60}}{F_{0}}\times 100$$

where %SR stands for percentage stress relaxation, F_0_ stands for peak force at 1 mm distance, and F_60_ stands for the force at the end of the 1 min relaxation process.

**Figure 8:**
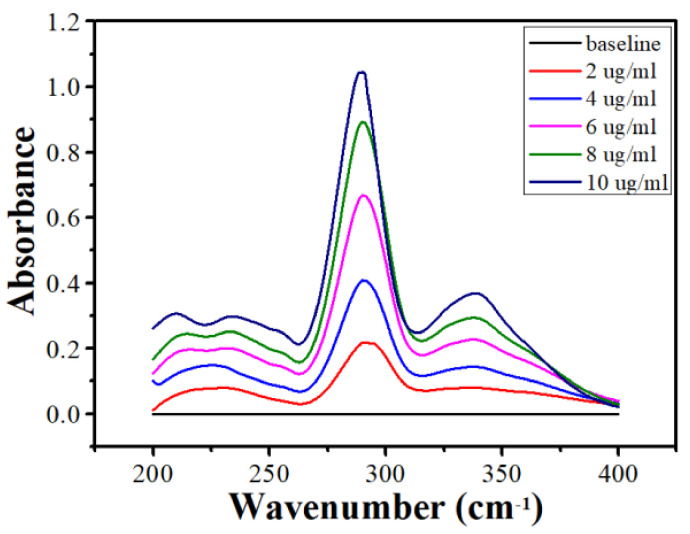
UV-vis spectra of the moxifloxacin HCI standard solutions.

The drug release from the bigels was studied in a dissolution apparatus (DS-8000, LabIndia analytical Instruments Pvt. Ltd., Mumbai, India). The dissolution apparatus was connected with a basket type (Type-I) sample holder attachment. The dissolution flask was filled with 400 mL of phosphate buffer (pH 6.8, 37 °C). The phosphate buffer solution was prepared by dissolving 28.20 g of disodium hydrogen phosphate and 11.45 g of potassium dihydrogen phosphate in 1000 mL double distilled water. Then, 1 g of the bigels was inserted into the basket. The basket was then rotated at a speed of 100 RPM. Next, at a specified time period (5, 15, 30, 45, 60, 90, 120, 150, and 180 min), 5 mL of the dissolution media was withdrawn for further analysis. The withdrawn dissolution media was replaced with fresh dissolution media. Thereafter, at the end of the study, the withdrawn dissolution media was analyzed in a UV-visible spectrometer (Shimadzu 3600 UV-VIS-NIR, Kyoto, Japan). The analysis was conducted at a wavelength of 290 nm to determine the concentration of the drug released into the dissolution media. The UV-vis spectra of the standard moxifloxacin HCI solution is compiled in Figure 8.

## Figures and Tables

**Table 1:** Color parameters of bigels.

Sample	L *	a *	b *	WI	YI
BG0	98.67 ± 0.96	−5.74 ± 1.16	19.06 ± 5.70	80.02 ± 5.77	27.60 ± 8.49
BG1	99.07 ± 0.19	−6.64 ± 1.40	19.80 ± 4.57	79.10 ± 4.78	28.55 ± 6.64
BG2	97.76 ± 2.03	−6.16 ± 2.67	23.92 ± 1.38	75.20 ± 1.09	34.95 ± 2.68
BG3	98.19 ± 1.17	−6.12 ± 1.45	22.13 ± 6.74	76.97 ± 6.82	32.20 ± 6.06
BG4	98.81 ± 0.37	−7.05 ± 0.44	21.97 ± 2.11	76.90 ± 2.12	31.77 ± 3.15

**Table 2:** Stress relaxation parameters of bigel formulations.

Formulations	F0	F60	%SR
BG0	205.37 ± 26.95	78.85 ± 8.09	61.60 ± 2.43
BG1	1349.64 ± 133.67	739.42 ± 105.43	45.21 ± 3.00
BG2	1569.40 ± 219.49	877.00 ± 77.08	44.18 ± 2.94
BG3	2548.71 ± 106.68	1123.40 ± 25.01	55.92 ± 2.60
BG4	2186.90 ± 328.30	971.89 ± 79.98	55.55 ± 3.45

**Table 3:** Korsmeyer-Peppas model drug release parameters.

Formulations	Model Parameters		
	K	n	R^2^
BG0D	2.294 ± 0.037	0.267 ± 0.016	0.995
BG1D	2.186 ± 0.188	0.308 ± 0.017	0.993
BG2D	3.045 ± 0.227	0.256 ± 0.007	0.988
BG3D	6.023 ± 1.726	0.193 ± 0.050	0.987
BG4D	4.362 ± 0.394	0.232 ± 0.020	0.992

**Table 4:** Summary of the key parameters.

Formulations	Mean Droplet Size	%SR	Diffusion Factor (K)
B0	22.42 ± 0.42	61.60 ± 2.43	2.294 ± 0.037
B1	20.53 ± 0.16	45.21 ± 3.00	2.186 ± 0.188
B2	20.45 ± 0.53	44.18 ± 2.94	3.045 ± 0.227
B3	20.30 ± 0.14	55.92 ± 2.60	6.023 ± 1.726
B4	20.45 ± 0.41	55.55 ± 3.45	4.362 ± 0.394

**Table 5:** Composition of the bigels.

Code	Hydrogel (g)	dp-CNC (mg)	Oleogel (g)	Moxifloxacin HCl (% *w*/*w*)
BG0	25	-	75	-
BG1	25	5	75	-
BG2	25	10	75	-
BG3	25	15	75	-
BG4	25	20	75	-
BG0D	25	-	75	0.25
BG1D	25	5	75	0.25
BG2D	25	10	75	0.25
BG3D	25	15	75	0.25
BG4D	25	20	75	0.25

## Data Availability

Data are contained within the article.
